# Are Platelet-Rich Products Necessary during the Arthroscopic Repair of Full-Thickness Rotator Cuff Tears: A Meta-Analysis

**DOI:** 10.1371/journal.pone.0069731

**Published:** 2013-07-12

**Authors:** Qiang Zhang, Heng’an Ge, Jiaojiao Zhou, Biao Cheng

**Affiliations:** 1 Department of Orthopedics, Shanghai Tenth People's Hospital, Tongji University, School of Medicine, Shanghai, China; 2 First Clinical Medical College, Nanjing Medical University, Nanjing, China; University of Rochester, United States of America

## Abstract

**Background:**

Platelet-rich products (PRP) are widely used for rotator cuff tears. However, whether platelet-rich products produce superior clinical or radiological outcomes is controversial. This study aims to use meta-analysis to compare clinical and radiological outcomes between groups with or without platelet-rich products.

**Methods:**

The Pubmed, Embase, and Cochrane library databases were searched for relevant studies published before April 20, 2013. Studies were selected that clearly reported a comparison between the use or not of platelet-rich products. The Constant, ASES, UCLA, and SST scale systems and the rotator cuff retear rate were evaluated. The weighted mean differences and relative risks were calculated using a fixed-effects model.

**Results:**

Seven studies were enrolled in this meta-analysis. No significant differences were found for the Constant scale (0.73, 95% CI, −1.82 to 3.27, P = 0.58), ASES scale (−2.89, 95% CI, −6.31 to 0.53, P = 0.1), UCLA scale (−0.79, 95% CI, −2.20 to 0.63, P = 0.28), SST scale (0.34, 95% CI, −0.01 to 0.69, P = 0.05), and the overall rotator cuff retear rate (0.71, 95% CI, 0.48 to 1.05, P = 0.08). Subgroup analysis according to the initial tear size showed a lower retear rate in small- and medium-sized tears (0.33, 95% CI, 0.12 to 0.91, P = 0.03) after platelet-rich product application but no difference for large- and massive-sized tears (0.86, 95% CI, 0.60 to 1.23, P = 0.42).

**Conclusion:**

In conclusion, the meta-analysis suggests that the platelet-rich products have no benefits on the overall clinical outcomes and retear rate for the arthroscopic repair of full-thickness rotator cuff tears. However, a decrease occurred in the rate of retears among patients treated with PRP for small- and medium-sized rotator cuff tears but not for large- and massive-sized tears.

**Level of Evidence:**

Level II

## Introduction

Rotator cuff tears are one of the most commonly occurring disorders of the shoulder, and they have a significant effect on daily life due to loss of motion and strength. Approximately 17% to 50% of adults older than 60 years and 80% of adults older than 80 years may have rotator cuff pathologies [1], [2], [3]. Surgical interventions are always needed, but the reconstructed tendon to bone insertion site rarely heals. Instead, a retear rate of 30% to 94% was found after a single row rotator cuff repair [4], [5], with an even higher rate in massive tears or old patients. Although strategies such as the “transosseous-equivalent’’ suture-bridge technique have been developed over the past decade to enhance postoperative healing, the outcomes are far from satisfactory. A mechanically inferior fibrovascular tissue, rather than native fibrocartilage tissue, forms at the repair site, exposing the insertion site to high stresses and increasing the risk of failure [6], [7], [8]. Consequently, the biological augmentation of the rotator cuff tendon to bone repair has gained increasing interest.

Recently, numerous growth factors, such as the bone morphogenetic proteins (BMPs), basic fibroblast growth factor (bFGF), platelet-derived growth factor (PDGF), vascular endothelial growth factor (VEGF), insulin-like growth factor 1 (IGF 1), and transforming growth factor-b (TGF-b), were found to improve the proliferation and collagen secretion of tenocytes in vitro and to increase the biomechanical strength and accelerate the tendon-to-bone healing in vivo [9], [10], [11], [12], [13], [14], [15], [16], [17], [18]. At the same time, healing is a highly complex biological process involving the precise coordination of various growth factors. Platelet-rich products (PRP) is a whole blood fraction containing high platelet concentrations that, once activated, releases the various growth factors mentioned above, which participate in the tissue repair process [19]. Therefore, the application of growth factor mixtures in the form of platelet-rich products provides a promising future for tendon-bone insertion regeneration such as rotator cuff repair. In fact, ample basic science and animal data have shown the positive effects of PRP on tendon collagen deposition and tendon vascularization [20], [21], [22]. Many clinical studies have also shown promising results of PRP application for a variety of indications [23], [24], [25]. Additionally, a range of platelet preparations have been approved by the US Food and Drug Administration and have been made commercially available.

Nevertheless, few clinical studies with a high level of evidence have proved that these advantages can be translated into improvements in the clinical and radiological outcomes for rotator cuff tears. Controversy continues regarding the effect of PRP application. However, none of the previous studies involved a large number of patients, which may prevent the identification of any differences between the groups. Consequently, meta-analysis is suitable to solve this problem. A previous meta-analysis showed a low level of evidence, but it included all types of studies, including retrospective studies [26]. The present study aims to conduct a meta-analysis of level I and II evidence studies to investigate the clinical and imaging outcomes of PRP application during the arthroscopic repair of full-thickness rotator cuff tears.

Our hypothesis is that the arthroscopic repair of full-thickness rotator cuff tears with or without PRP application would not show any clinical or radiological differences.

## Methods

The Pubmed, Cochrane library, and Embase databases were searched independently by 2 investigators (Q.Z. and H.A.G.) to retrieve relevant studies published before January 1, 2013. The search criteria “rotator cuff”, “platelet rich plasma”, “PRP”, “platelet rich fibril matrix”, “PRFM” and “platelet” were used in text word searches. The “related articles” function was used to broaden the search. The reference lists of the selected articles were also manually examined to find relevant studies that were not discovered during the database searches. On April 20, 2013, the databases were searched again for additional studies.

### Inclusion criteria

1. Prospective studies of Level I or II evidence
2. Arthroscopic rotator cuff repair
3. Study comparing outcomes with and without PRP application
4. Greater than 12-month minimum follow-up
5. Follow-up examination presenting at least one of the following outcome measurements: ASES score, Constant score, UCLA scale, SST scale, and radiographic (MRI and/or USG) follow-up of repaired rotator cuffs

### Exclusion criteria

1. Retrospective study
2. Level III or IV evidence studies
3. Less than 12-month minimum follow-up
4. Studies only reporting outcomes after PRP application
5. Studies that included open or mini-open procedures
6. Studies involving partial thickness rotator cuff tears

### Data extraction

The data extraction of all variables and outcomes of interest and the assessment of methodological quality were performed independently by 2 readers (Q.Z. and H.A.G.). Disagreements were resolved through discussion and consensus. The methodological quality of the trials was assessed using the Cochrane Handbook for Systematic Reviews of Interventions 5.1.

### Outcomes

Both subjective and objective functional outcome measurements were used to evaluate the data. The Constant scale, American Shoulder and Elbow Surgeons scale (ASES), University of California at Los Angeles scale (UCLA) and Simple Shoulder Test scale (SST) were analyzed to determine the functional outcome. The Constant scale was compared at the time point of approximately 20 months post-operatively. The ASES scale, UCLA scale, SST scale, and radiological assessment were compared approximately 12 months post-operatively. The rotator cuff integrity was divided into integrity and retear. Subgroup analysis was performed according to the initial tear size.

### Statistical analysis

The statistical analysis was performed using Review Manager 5.1 (Cochrane Collaboration, Nordic Cochrane Centre, Copenhagen, Denmark). Continuous variables were analyzed using the weighted mean difference, whereas categorical dichotomous variables were assessed using relative risks (RRs). A P value <0.05 was considered to be statically significant, and 95% confidence intervals (CIs) were reported. Homogeneity was tested by the Q statistic (significance level at P<0.10) and the I^2^ statistic (significance level at I^2^>50%). A random-effects model was used if the Q or I^2^ statistic was significant; otherwise, a fixed-effects model was used. The presence of publication bias was assessed by a visual inspection of a funnel plot.

## Results

### Literature Search

**Figure 1:**
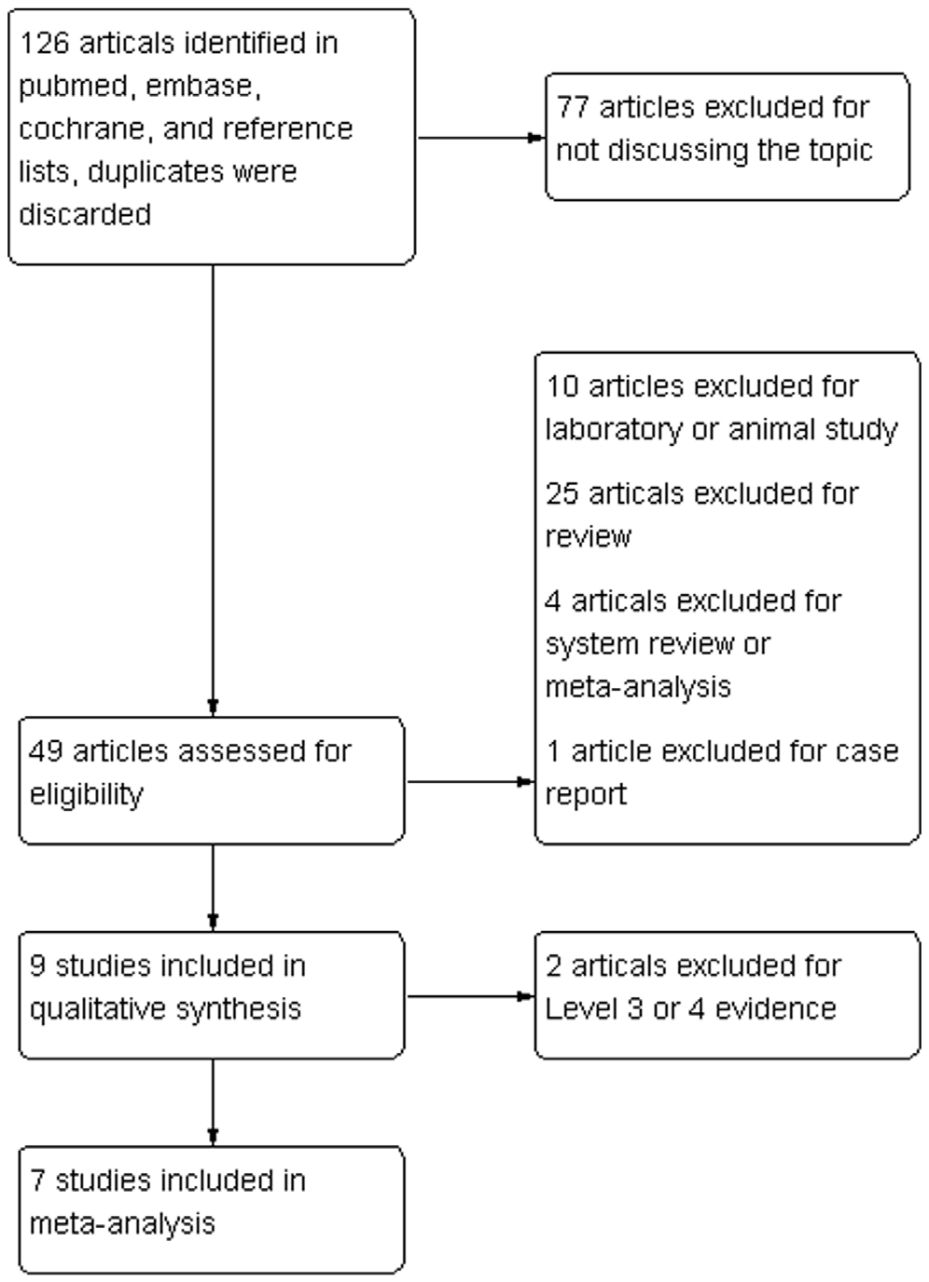
Search strategy flow diagram.

The initial literature search retrieved 126 relevant articles (duplicates were discarded). Seventy-seven articles were excluded for not investigating the topic after carefully screening the titles. Then, the abstracts were reviewed, and 40 articles were excluded (10 laboratory or animal studies, 25 reviews, 4 system reviews and meta-analyses, and 1 case report), which left 9 studies for further full publication review. Two studies were excluded for level III or IV evidence [27], [28]. Therefore, 7 studies matched the selection criteria and were suitable for this meta-analysis [29], [30], [31], [32], [33], [34], [35], with 6 being prospective randomized control trials and 1 being a prospective cohort study. The flow-diagram is shown in Figure 1. A total of 379 patients (185 for PRP application and 194 for control) were enrolled in the studies. The key characteristics of the included studies are summarized in Table 1. All the studies involved patients with reparable full-thickness rotator cuff tears and who were followed for at least 12 months. Among the included studies, the Constant scale, ASES scale, UCLA scale, SST scale, and radiological (MRI) assessment were matched in 3 studies. A review of the data extraction revealed 100% agreement between the 2 reviewers.

**Table 1:** The characteristics of the included studies.

Study	Country	Study Design	Patients	Intervention	Sample Size(PRP VS no PRP)	Mean Follow-up	Matching Outcome Measures
Roberto Castricini 2011 ^6^	Italy	RCT Level 1	reparable full-thickness rotator cuff tears	double row technique with or without PRFM	43 VS 45	16 months	Constant
Pietro Randelli 2011 ^28^	Italy	RCT Level 1	reparable full-thickness rotator cuff tears	single row technique with or without PRP	22 VS 23	24 months	Constant,UCLA,SST,MRI
Chris Hyunchul Jo 2012 ^20^	Korea	Prospective Cohort study Level 2	reparable full-thickness rotator cuff tears	suture bridge double row technique with or without PRP	19 VS 23	18.94±1.63 VS 20.3±1.89 months	ASES,Constant,UCLA,SST,MRI
Scott A. Rodeo 2012 ^29^	USA	RCT Level 2	repairable full-thickness rotator cuff tears	single or double row techniques with or without PRFM	19 VS 22	12 months	ASES
Stefano Gumina 2012 ^13^	Italy	RCT Level 1	reparable large full-thickness rotator cuff tears	single row technique with or without platelet-leukocyte membrane	39 VS 37	13 months	Constant,SST,MRI
Stephen C. Weber 2012 ^36^	USA	RCT Level 1	reparable full-thickness rotator cuff tears	single row technique with or without PRFM	29 VS 30	12 months	ASES,UCLA

**Figure 2:**
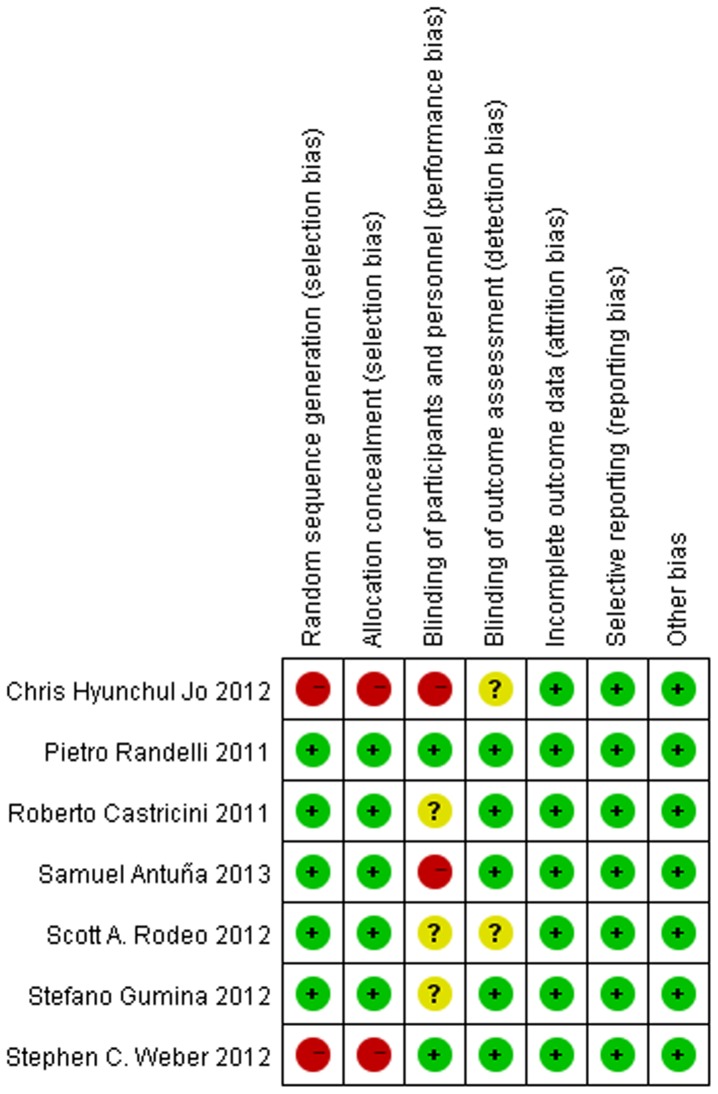
The methodological quality of the included studies.

Figure 2 summarizes the methodological quality of the studies. Six of the studies were RCTs with a high level of methodological quality, and the seventh was a prospective cohort study. The methodological bias of these studies was low.

### Main analysis

**Figure 3:**
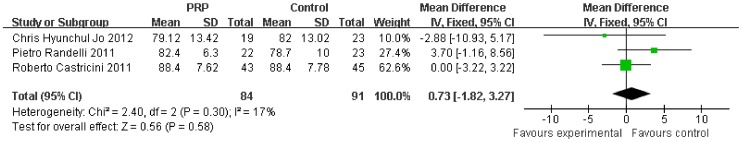
Difference in the Constant scale.

**Figure 4:**
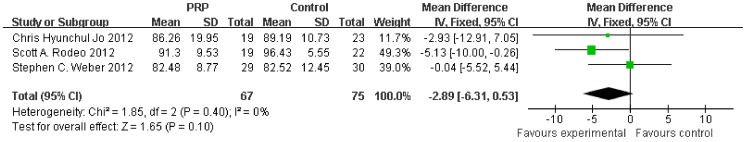
Difference in the ASES scale.

**Figure 5:**
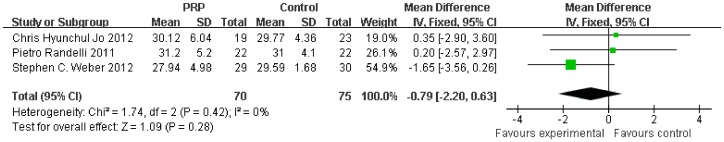
Difference in the UCLA scale.

**Figure 6:**
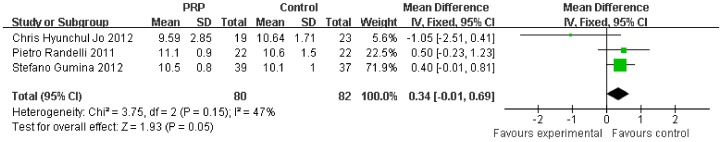
Difference in the SST scale.

**Figure 7:**
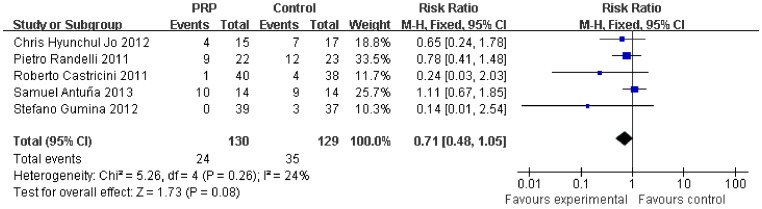
Difference in the rotator cuff retear rate.

Table 2 summarizes the outcomes of the meta-analysis. No significant difference was found between the PRP group and the control group for the Constant scale (0.73, 95% CI, -1.82 to 3.27, P = 0.58) (Figure 3), ASES scale (−2.89, 95% CI, −6.31 to 0.53, P = 0.1) (Figure 4), UCLA scale (−0.79, 95% CI, −2.20 to 0.63, P = 0.28) (Figure 5), SST scale (0.34, 95% CI, −0.01 to 0.69, P = 0.05) (Figure 6), or overall rotator cuff retear rate (0.71, 95% CI, 0.48 to 1.05, P = 0.08) (Figure 7).

**Table 2:** Meta-analysis of the outcomes of interest.

Outcomes of interest	No. of Studies	Participants	Overall effect		Heterogeneity	
			RR or WMD (95% CI)	P Value	I^2^, % (95% CI)	P Value
Constant	3	175	0.73 [−1.82, 3.27]	0.58	17%	0.3
ASES	3	142	−2.89 [−6.31, 0.53]	0.1	0%	0.4
UCLA	3	145	−0.79 [−2.20, 0.63]	0.28	0%	0.42
SST	3	162	0.34 [−0.01, 0.69]	0.05	47%	0.15
retear rate	5	259	0.71 [0.48, 1.05]	0.08	24%	0.26
retear rate (Small-Medium)	3	130	0.33 [0.12, 0.91]	0.03	0%	0.94
retear rate (Large-Massive)	4	129	0.86 [0.60, 1.23]	0.42	11%	0.34

**Figure 8:**
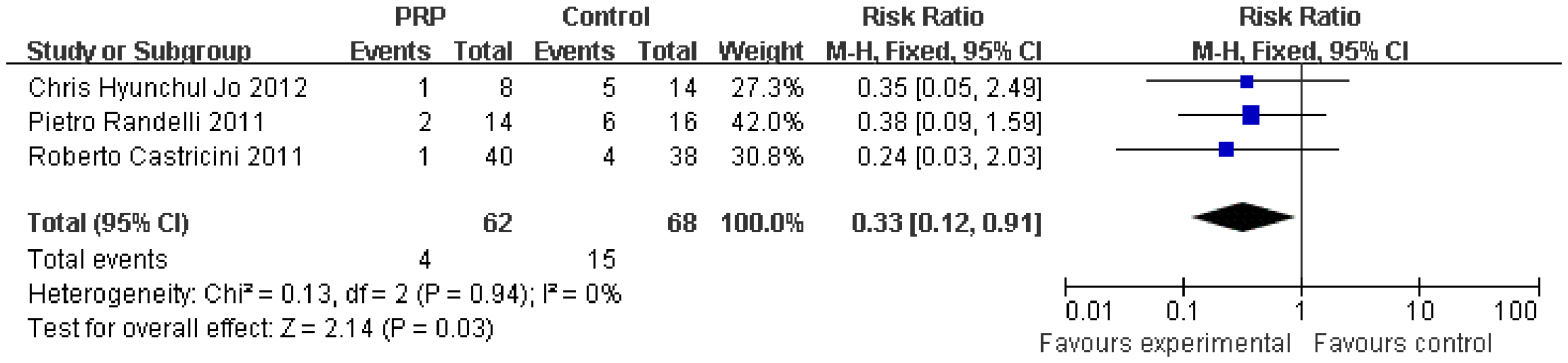
Difference in the retear rate of small- and medium-sized rotator cuff.

**Figure 9:**
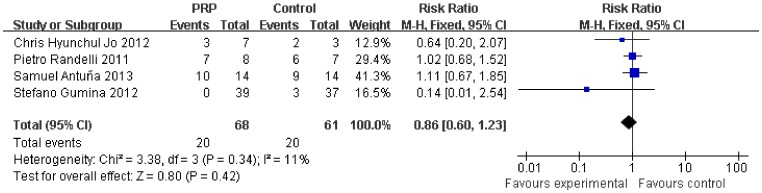
Difference in the retear rate of large- and massive-sized rotator cuff.

Subgroup analysis according to the initial tear size was available for the retear rate. The initial tear size was divided into two groups (small-medium and large-massive). The retear rate was lower after PRP application in the small-medium tears (0.33, 95% CI, 0.12 to 0.91, P = 0.03) (Figure 8), but no significant difference was found in the large-massive tears (0.86, 95% CI, 0.60 to 1.23, P = 0.42) (Figure 9).

No significant heterogeneity was found among these studies (Constant, P = 0.30, I^2^ = 17%; ASES, P = 0.40, I^2^ = 0%; UCLA, P = 0.42, I^2^ = 0%; SST, P = 0.15, I^2^ = 47%; retear rate, P = 0.48, I^2^ = 0%; small-medium retear rate, P = 0.94, I^2^ = 0%; large-massive retear rate, P = 0.34, I^2^ = 11%).

### Publication bias

The funnel plots demonstrated no visual evidence of publication bias.

## Discussion

The treatment of rotator cuff tears has progressed rapidly over the last several years. New techniques such as arthroscopy, improved fixation devices, and several fixation methods have reduced the pain and improved the function of the patients. Biological methods have also been investigated, as the outcomes are far from satisfactory, and among these methods, platelet-rich plasma has gained increasing interest.

PRP, most simply defined as a sample of autologous blood with high concentrations of platelets that contains various growth factors, can be applied by either direct injection or the physical application of a PRP matrix scaffold to the target tissues [36], [37]. PRP is thought to augment the natural healing process as it can increase the concentration of growth factors at the site of the injury. PRP application showed great benefits in basic science, animal models, and some low level evidence studies, whereas few clinical studies with a high level of evidence reported similar results. Controversy is still ongoing regarding the clinical efficacy of platelet-rich products.

With the present meta-analysis of levels I and II evidence prospective control studies, we were able to support our primary hypothesis that there were no differences in the overall retear rates or functional outcomes (Constant scale, ASES scale, UCLA scale, and SST scale) among patients who were administered platelet-rich products during the arthroscopic repair of full-thickness rotator cuff tears and those who were not. However, there was a decrease in the rate of retears observed among patients treated with PRP in the setting of small- and medium-sized rotator cuff tears but no change in the setting of large- and massive-sized tears.

Interestingly, the basic and animal studies showed promising results, whereas the clinical investigation reported similar or even negative results [32]. The application of growth factor mixtures in the form of PRPs provides an autologous source of useful anabolic agents. However, the increased levels of the various growth factors are not well controlled. For example, excessive exposure to TGF-b, with its potential for exuberant fibrosis, is a real possibility. Additionally, the effect of PRP application suffers greatly from the limited residence time. Most importantly, thrombin from the fibril matrix has been shown to accelerate the release of growth factors, which means that this release cannot be sustained long term. Thrombin has been shown to significantly decrease the efficacy of PRP in bone grafting [38]. However, various platelet-rich products may also influence the effect, including the volume of blood, single- versus double-spin cycles, centrifuge rates, the need for an activator, white blood cell concentrations, and the final platelet and growth factor concentrations.

Moreover, although PRP is obtained from the patient's own blood, these products are not without risk. The preparation of the PRP increases the risk of infection, even though it is performed with sterile handling. Additionally, pregnancy, thrombocytopenia, anticoagulation therapy, active infection, tumor, or metastatic disease may also limit the application of PRP. Increased postoperative stiffness has also been a concern as PRP significantly increases fibrosis [31]. The absorption of the PRFM may also create another gap in the footprint [39]. Otherwise, the cost-effectiveness is also a problem as the cost of the platelet-rich products is high.

Given these factors, we demonstrated that the platelet-rich products showed no benefits on overall clinical and radiological outcomes, and potentially even showed disadvantages for full-thickness rotator cuff tears. However, there was a decrease in the rate of retears among patients treated with PRP in the setting of small- and medium-sized rotator cuff tears but no change in the setting of large- and massive-sized tears. As the studies included were of high methodological quality and had no significant heterogeneity and as this meta-analysis was supported by a relatively larger number of patients, its conclusion was persuasive and can guide future clinical work.

However, some limitations exist in this meta-analysis. First, a meta-analysis according to the initial tear size for the functional outcomes could not be performed. Because there was a lower retear rate for small- and medium- sized rotator cuff tears, determining whether there were better functional outcomes was important. Second, we did not investigate the clinical and radiological outcomes in a short follow-up as PRP was considered to accelerate the healing process. Third, we used random control trials with level I evidence to increase the sample size and the power of our analysis, which may introduce bias to the results. Fourth, the above-mentioned clinical heterogeneity was high. Among the six studies, 3 were from Italy, 2 were from the USA, and 1 was from Korea, which means that there was a great difference among the involved patients. Additionally, different studies employed different repair techniques, such as the single and double row techniques. The tear size ranged from small to large full-thickness rotator cuff tears. Furthermore, different PRP products were used among the studies, including the volume of blood, single- versus double-spin cycles, centrifuge rates, the need for an activator, white blood cell concentrations, and the final platelet and growth factor concentrations. Finally, although we included 7 studies, the number of the patients included who matched outcomes with regard to the time point were small. The overall sample size may not have had adequate power to detect smaller differences.

In the future, multicenter prospective randomized control trials with large samples and various subgroups according to tear size are needed. Although many limitations exist, this study is still powerful enough to guide clinical work.

## Conclusion

In conclusion, the meta-analysis suggests that the platelet-rich product has no benefits on the overall clinical outcomes and retear rate for the arthroscopic repair of full-thickness rotator cuff tears. However, there was a decrease in the rate of retears among patients treated with PRP in the setting of small- and medium-sized rotator cuff tears but no change in the setting of large- and massive-sized tears.

## Supporting Information

Table S1 **Completed PRISMA checklist.** Table S1 presents the completed PRISMA checklist for the meta-analysis.
(DOC)
